# High-capacity multiview display with large viewing angle via orbital angular momentum-encoded nanograting arrays

**DOI:** 10.1515/nanoph-2025-0433

**Published:** 2025-11-03

**Authors:** Yiqi Ye, Hang Su, Yuetian Jia, Baoli Li, Min Gu, Xinyuan Fang

**Affiliations:** School of Artificial Intelligence Science and Technology, 47863 University of Shanghai for Science and Technology, Shanghai, 200093, China; Institute of Photonic Chips, University of Shanghai for Science and Technology, Shanghai, 200093, China

**Keywords:** 3D display, multiview, orbital angular momentum, large field of view, high-capacity, forked nanograting array

## Abstract

Three-dimensional (3D) displays reconstruct spatial light fields, providing immersive stereoscopic experiences with depth perception. Multiview 3D displays are particularly attractive, delivering multiple perspective images to different spatial positions for glasses-free multi-user observation and continuous motion parallax. However, achieving both high-capacity information encoding and a large field-of-view (FOV) remains challenging. Here, we propose a high-capacity, large-FOV holographic multiview 3D display by integrating orbital angular momentum (OAM) multiplexing with forked nanograting arrays fabricated via two-photon lithography (TPL). A 3 × 3 hologram array is loaded onto a spatial light modulator (SLM), with each sub-hologram encodes four orthogonal OAM modes, enabling parallel high-capacity information storage. Each OAM channel is diffracted by the corresponding forked nanograting array into multiple discrete directions (experimentally verified up to nine), effectively expanding the accessible viewing range. A dual dynamic control mechanism allows real-time hologram refresh on the SLM and selective switching of different OAM-encoded image sets without computational latency. Experiments under 532 nm illumination successfully reconstruct eight independent 3D scenes with nine viewpoints across a 30° field of view, achieving an average structural similarity index (SSIM) of ∼0.81 with negligible crosstalk. This work establishes a reconfigurable, high-throughput, large-FOV multiview 3D display framework, with potential for portable AR/VR devices, holographic communication and medical surgical navigation.

## 1 Introduction

Three-dimensional (3D) display technology [1], [2], [3], [4] has emerged as a key frontier in modern display research, owing to its ability to present rich depth information and deliver immersive stereoscopic experiences that surpass the inherent limitations of conventional two-dimensional displays. Among various approaches, multiview 3D display is particularly appealing [5], [6], [7], [8], [9], as it enables distinct viewpoint images to observers at different positions and enables motion parallax perception simultaneously without auxiliary devices. This technique has already been applied in fields such as medical imaging [10], virtual/augmented reality (VR/AR) interactions [11], [12] and high-precision industrial inspection. Current mainstream implementations of multiview 3D display primarily include directional backlighting [13], [14], lenticular lens arrays [15], [16], [17] and metasurface-based beam deflection [18], [19], which have demonstrated progress in reconstructing multi-perspective image. However, the growing complexity and information density of modern digital content place increasing demands on the throughput of multiview 3D display systems. At the same time, ensuring smooth transitions between adjacent viewpoints and maintaining continuous depth perception without visual constraints or discomfort necessitates a large field of view (FOV). Consequently, achieving a large FOV alongside high-throughput multiview capability in a single 3D display system remains a critical challenge for practical applications.

Holography, as a genuine approach for multiview 3D displays, enables the capture of complete light information and the reconstruction through precise wavefront modulation [20], [21]. In particular, holographic multiplexing encodes multiple information channels within a single hologram by exploiting different physical degrees of freedom of light, such as wavelength [22], [23], [24], polarization [25], [26], [27] or frequency [28]. Individual channels can then be selectively retrieved through distinct diffraction orders with minimal crosstalk. Among the various multiplexing strategies, orbital angular momentum (OAM) beams offer a compelling solution due to their intrinsic orthogonality and theoretically unbounded dimensionality [29], [30], [31], [32], [33]. By assigning distinct OAM modes to separate channels, multiple information channels can be simultaneously reconstructed and dynamically controlled [34], [35], offering a feasible pathway toward high-capacity multiview 3D display systems.

To expand the FOV, several optical strategies have been explored. One widely used strategy is spatial light modulator (SLM) stitching, in which multiple SLMs are tiled to enlarge the effective aperture and increase the diffraction angle, thereby broadening the FOV [36]. However, this method faces challenges such as complex alignment, high cost and increased system size. Liquid crystal grating techniques offer dynamic tunability, yet their inherently slow response speed and relatively low diffraction efficiency limit performance in high-speed and high-resolution applications [37]. Although these strategies have advanced the trade-offs among large FOV, high information capacity and system simplicity, a fully integrated solution that simultaneously fulfills all these requirements remains lacking, leaving a critical gap for realizing practical high-capacity, large-FOV multiview 3D displays.

In this work, we propose a high-capacity multiview 3D display that achieves both a large viewing angle and high throughput by combining OAM multiplexing with a nanograting-array expansion scheme. In our system, a tiled 3 × 3 OAM-multiplexing hologram array is loaded on an SLM, which operates in conjunction with a forked nanograting array. Each OAM channel is diffracted into multiple discrete directions by the forked grating array (up to nine experimentally), thereby enabling parallel multi-channel reconstruction across an effectively expanded viewing range. The system incorporates two dynamic control mechanism: updating the hologram arrays on the SLM refreshes the displayed content in real time, while replacing the forked nanograting array decodes different OAM-encoded image sets without computational latency. This hybrid design leverages the orthogonality of OAM modes for high-capacity encoding and the geometric functionality of forked nanograting array [38], [39] for efficient FOV broadening. Experiments under 532 nm illumination demonstrate the successful reconstruction of eight independent 3D scenes across two OAM-multiplexing holograms and four OAM channels within a 30° FOV, with each scene comprising nine viewpoints. The system achieves an average structural similarity index (SSIM) of ∼0.81, confirming high fidelity and negligible crosstalk [40]. This approach not only overcomes the limitations of existing holographic multiview 3D displays but also establishes a paradigm for reconfigurable, high-capacity and large-FOV display systems, with broad potential in portable AR/VR devices [41], [42], holographic communication, glasses-free multiview display [43] and medical surgical navigation.

## 2 Design and principles

### 2.1 Multiview 3D holographic display architecture

**Figure 1:**
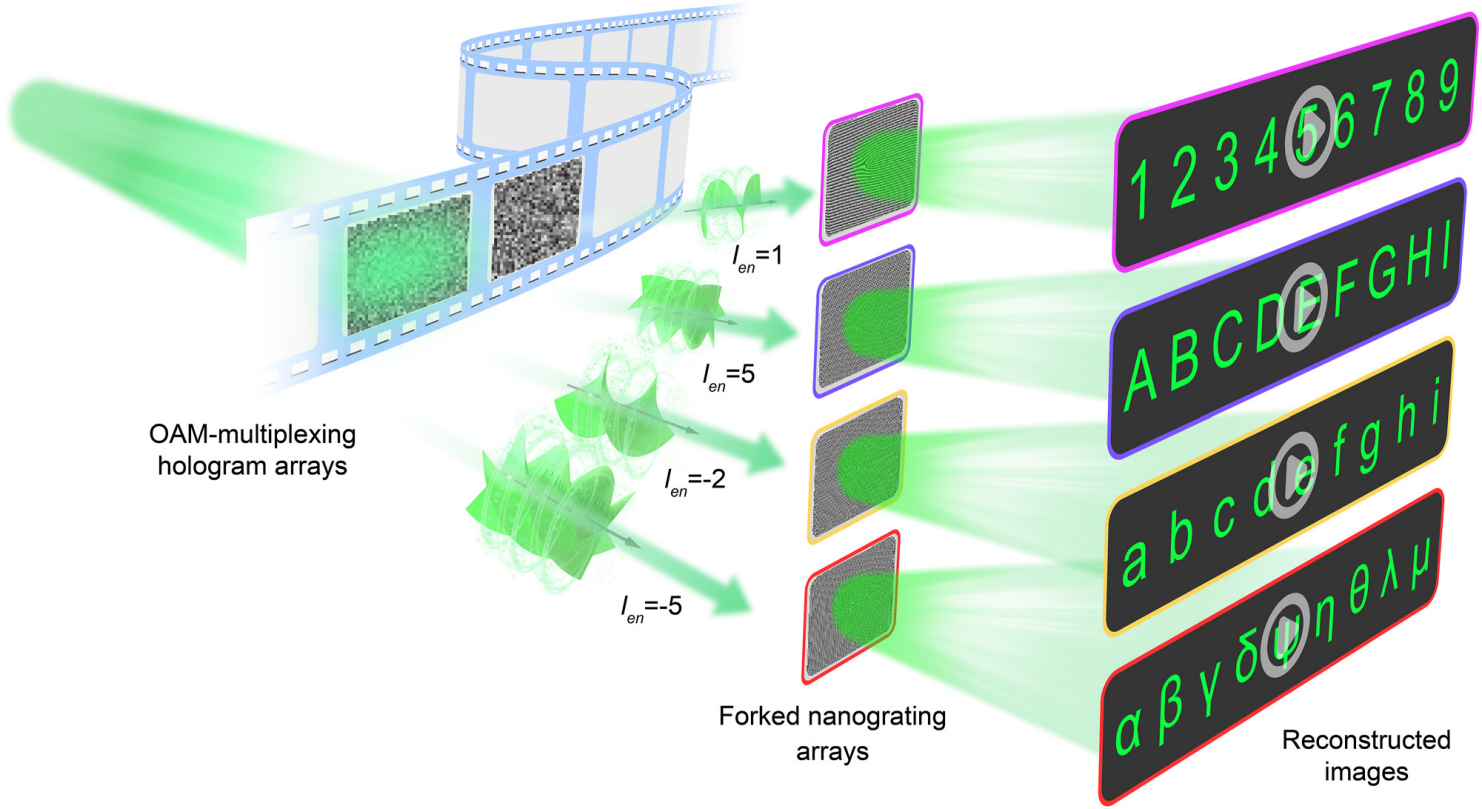
Schematic of the multiview 3D holographic display. Light modulated by an SLM in reflected onto TPL-fabricated forked nanograting array placed directly behind it, their tunable periods and secondary diffraction expand the SLM FOV from 8° to 30°. A predesigned 3 × 3 OAM-multiplexing hologram array enables dynamic switching (by swapping the forked nanograting array or the loaded OAM-multiplexing hologram array). The matching 3 × 3 forked nanograting array yields nine diffraction orders, each reconstructing an independent image for multi-user viewing.

Figure 1 schematically illustrates the novel multiview 3D holographic display architecture based on the integration of grating array and OAM-multiplexing techniques. The system employs a clear optical path for large-FOV image reconstruction, where incident light first interacts with an SLM loaded with holographic patterns, and then reflects directly onto the two-photon lithography (TPL)-fabricated forked nanograting array(positioned immediately behind the SLM). The TPL-fabricated forked nanograting array play a dual role in expanding FOV: their tunable grating periods match the spatial frequency of the SLM’s output light, while their secondary diffraction effect breaks the diffraction angle limitation of conventional hologram reconstruction (Supplementary Note 1, Supplementary Figure S1, Supplementary Information). This synergy enables the system to extend the intrinsic FOV from 8° to 30°, a critical improvement that overcomes the inherent pixel-size-induced FOV constraint of conventional SLMs.

To realize high-capacity multiview 3D display, we first pre-design the holographic content by generating a set of large-FOV OAM-multiplexing sub-holograms, with each sub-hologram encoding independent image information for different channels and views. Through spatial multiplexing technology, these sub-holograms are precisely arranged into the corresponding positions of a 3 × 3 large-FOV OAM-multiplexing hologram array, ensuring each sub-hologram retains independent channel encoding capability while integrating into a single array. When this 3 × 3 array is loaded onto the SLM, dynamic content switching can be achieved in two ways: one is by replacing the TPL-fabricated forked nanograting array (without reconfiguring the SLM), and the other is by switching the OAM-multiplexing hologram array loaded on the SLM. Finally, the reflected light from the SLM is coupled into the matching 3 × 3 forked nanograting array, which diffracts the light into nine distinct diffraction orders. Each diffraction order reconstructs a sub-hologram, collectively generating multiple large-FOV, high-capacity multiview displays suitable for simultaneous multi-user viewing.

### 2.2 Design principle of large-FOV multiview 3D display

**Figure 2:**
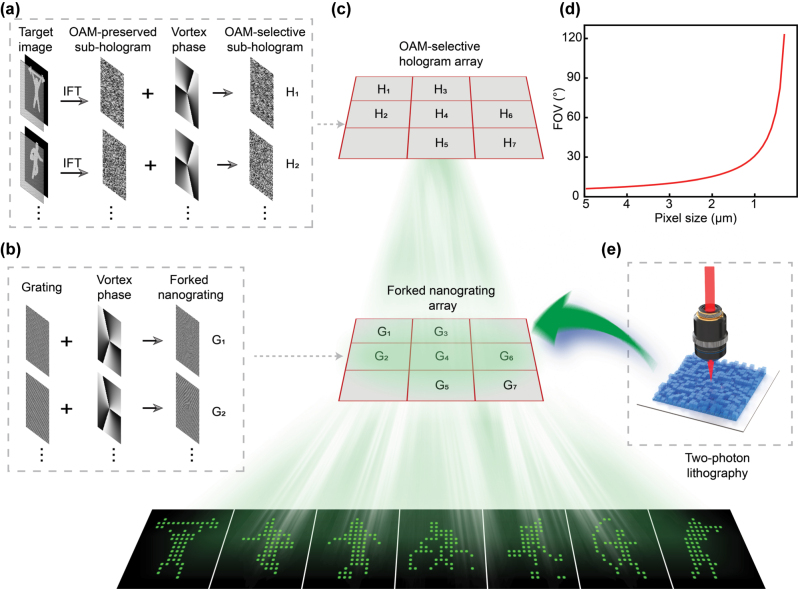
Design principle of the large-FOV, multiview 3D display. (a) Design framework of large- FOV OAM-selective sub-hologram. (b) Design principle of the forked nanograting. (c) Reconstruction process of large-FOV OAM-selective holograms. (d) The relationship between pixel size and the FOV. (e) Schematic diagram of forked nanograting array fabricated by TPL.

To realize large-FOV multiview 3D displays, a OAM-selective hologram is first designed, as illustrated in Figure 2a. OAM-preserved holograms corresponding to different target images are generated via iterative Fourier transform, and the transform is specifically performed on the product of the target image and a sampling array in the image plane. To realize OAM selectivity for large-FOV holography, a helical phase plate with a topological charge *l*_en_ = 3 is encoded into the OAM-preserved holograms. This process is mathematically described by Eq. (1):

(1) $$\begin{array}{ll} H\left(x,y\right) & =\overset{U}{\underset{u=1}{\sum }}\overset{V}{\underset{v=1}{\sum }}O\left(u,v\right)\exp\left[j2\pi \left(ux+vy\right)\right]\\ & \;\cdot \exp\left(jl_{en}\varphi \left(x,y\right)\right) \end{array}$$

Here, (*x*, *y*) and (*u*, *v*) denote the coordinates in the hologram plane and the image plane, respectively; *O* (*u*, *v*) represents the target image; *l*_en_ is the topological charge of the encoded OAM mode; and *φ*(*x*, *y*) is the azimuthal angle in polar coordinates. For the design of the forked nanograting array critical to large-FOV multiview 3D display, as illustrated in Figure 2b, two key parameters must be controlled: the topological charge (*l*) and the position of diffraction orders in the spatial frequency domain. To decode the encoded information, a topological charge of *l*_re_ = −3 is inscribed into the forked nanograting, resulting in the forked nanograting. The transmission function of the *m*-th fork-shaped grating in the nanograting array is expressed by Eq. (2):

(2) $$f_{m}\left(\varphi \right)=A_{m}\exp\left(il_{re}\varphi \right)\exp\left(ig_{m}\right)$$

where *l*_re_ is the topological charge of the decoder, and *A*_m_ denotes the amplitude weighting coefficient of the elementary grating function *g*_m_. Notably, both SLM loading and grating fabrication rely on phase-only modulation, and the corresponding phase-only form of the transmission function is given by Eq. (3):

(3) $$g_{m}\left(\varphi \right)=\exp\left[ip_{\text{m}}\left(\varphi \right)\right]$$

where *p*_m_(*φ*) can be expressed as:

(4) $$p_{m}\left(\varphi \right)=\text{Re}\;\left\{-i\ln\left[B_{m}\exp\left(il_{re}\varphi \right)\exp\left(ig_{m}\right)\right]\right\}$$

where *B*_m_ is a decisive factor for *p*_m_(*φ*), and Re (·) denotes the real part operator. The above steps outline the design process for the sub-holograms matching the forked nanograting array. As shown in Figure 2c, to construct a modulation system supporting seven diffraction orders, the pre-designed OAM-selective and forked nanograting are embedded into the corresponding positions of their respective arrays through spatial multiplexing, ensuring precise alignment between the hologram array and nanograting array. In 3D display systems, the maximum diffraction angle (*θ*) is jointly determined by the pixel size (*p*) and the wavelength (*λ*), and can be described by Eq. (5):

(5) $$\theta =\arcsin\left(\frac{\lambda }{2p}\right)$$

Based on this equation, the relationship of maximum diffraction angle and pixel size is illustrated in Figure 2d. To address the FOV limitation in 3D display, we reduce the pixel size of the forked nanograting to expand the system’s angular range. In practice, this is realized using TPL (Figure 2e), which provides nanometer-scale spatial resolution and ultra-fine 3D micro/nanofabrication capabilities. This technique overcomes the pixel sizes constraints of conventional holographic devices, thereby enabling the experimental realization of large-FOV multiview 3D display.

## 3 Results and discussion

### 3.1 Experimental characterization of large-FOV multiview 3D display

**Figure 3:**
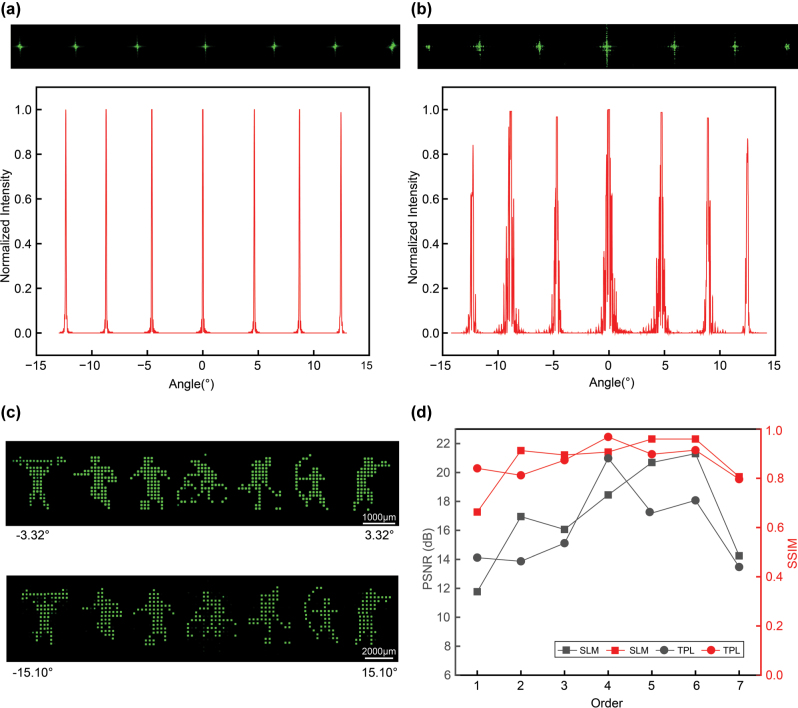
Experimental characterization of the optically digitalized large-FOV, multiview 3D display. (a) Simulation results of various diffraction orders and their normalized intensity distributions. (b) Experimental results of various diffraction orders and their normalized intensity distributions. (c) Large-FOV OAM-selective hologram experimental results with forked nanograting array of varying pixel sizes. (d) Large-FOV OAM-selective hologram experimental results with forked nanograting array of varying pixel sizes.

The simulation results are shown in Figure 3a, which indicate that the designed forked nanograting array can generate a series of diffraction orders aligned along the same horizontal axis, with uniform normalized intensities across these orders. By contrast, the experimental results reveal a certain degree of intensity reduction at wider diffraction angles. Gaussian illumination typically causes weaker intensity at the periphery and overexposure at the center of reconstructed patterns. To mitigate this effect, we employed a collimated homogenization converter which redistributes the incident beam energy uniformly. Consequently, multiple diffraction orders with relatively uniform normalized intensities are also obtained in the experiment (Figure 3b). Next, we experimentally validated the large-FOV multiview 3D display. Seven cartoon figures are chosen as target images. Using the algorithm described in Figure 2, seven large-FOV OAM-selective sub-holograms are calculated and then spatially multiplexed into an OAM-selective hologram array. Similarly, the forked nanograting was multiplexing to form the forked nanograting array.

In the experimental setup, the OAM-selective hologram array was conjugated to the forked nanograting array plane, ensuring each sub-hologram was precisely aligned with its designated modulation region. Two different devices were employed for image reconstruction: an SLM with a pixel size of 3.74 μm and a forked nanograting array fabricated by TPL with a pixel size of 1 μm. The reconstructed results are shown in Figure 3c. The first row presents the reconstructions obtained using the SLM-based forked nanograting array, where the seven figures were clearly reproduced but confined within a narrow FOV ranging from −3.32° to 3.32°. The second row shows results from the TPL-fabricated forked nanograting array, where the seven figures were also clearly reconstructed, while the FOV was significantly expanded to −15.1° to 15.1°. The procedure for calculating the field of view is detailed in Supplementary Note 2 (Supplementary Information).

Furthermore, a quantitative analysis of the reconstructed image quality under the two different pixel size conditions is presented in Figure 3d. Compared with the target images, both approaches yielded an average SSIM of 0.87, with peak signal-to-noise ratios (PSNR) of 17.07 dB and 16.12 dB, respectively. These results demonstrate that, compared with the conventional approach of loading gratings onto the SLM, our method achieves a substantially enlarged FOV for multiview 3D display while maintaining high image fidelity. This confirms the feasibility of the proposed large-FOV multiview 3D display system based on TPL-fabricated forked nanograting array. To further explore and validate the practical application of the system, we perform multi-view reconstructions of 3D holographic objects (Supporting Figure S3, Supporting Information).

### 3.2 Experimental demonstration of the high-capacity large-FOV multiview 3D display

**Figure 4:**
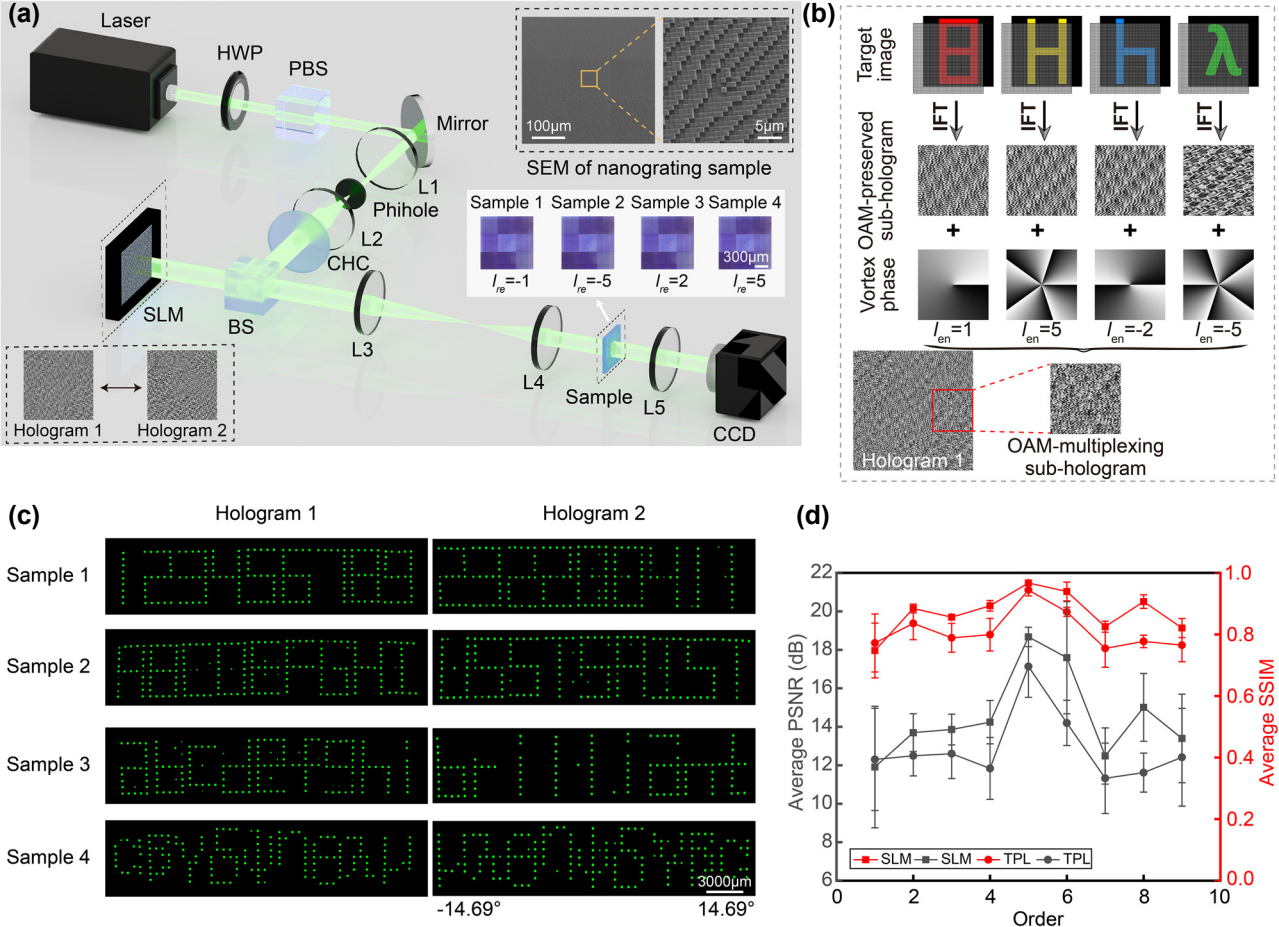
Experimental characterization of the high-capacity large-FOV multiview 3D display. (a) Schematic illustration of the optical setup. (b) Design and encoding process of large-FOV OAM-multiplexing holograms. (c) Results of two large-FOV OAM-multiplexing holograms with four identical forked nanograting arrays. (d) Large-FOV OAM-multiplexing hologram experimental results with forked nanograting array of varying pixel sizes.

Leveraging the OAM sensitivity of large-FOV OAM-selective holograms, we propose a high-capacity large-FOV multiview 3D display scheme. Here, 36 target images (Arabic numerals, uppercase letters, lowercase letters and Greek letters) are distributed across nine diffraction orders, with each group further encoded into four independent information channels. Each diffraction order corresponds to a distinct view, thereby enabling a multiview 3D display. Figure 4b illustrates the encoding process of one multiplexing sub-hologram. First, the target images ‘8, H, h, and λ’ are encoded into four corresponding large-FOV OAM-preserved sub-holograms using the iterative Fourier transform algorithm; Then, superimposed with different spiral phases (*l*_en_ = 1,5, −2,-5, the selection of *l*_en_ is detailed in the Supplementary Figure S2 (Supplementary Information)). to form the large-FOV OAM-multiplexing sub-hologram. Other diffraction orders can be obtained following the same procedure. Finally, all multiplexing sub-holograms are spatially combined to generate the complete large-FOV OAM-multiplexing hologram array.

Furthermore, we achieved digital implementation of high-resolution fork nanograting array using TPL. A forked nanograting array sample with the feature size of 1 μm × 1 μm and total size of 1,200 μm × 1,200 μm was fabricated. Microscopy and SEM images of the sample are shown in the upper-right inset of Figure 4a. What’s more, optical characterization of the digitally encoded large-FOV OAM-multiplexing hologram was carried out using the experimental setup in Figure 4a. As shown in Figure 4a, a 532 nm laser source (CNIlaser MSL-FN-532) was used, with a half-wave plate (HWP, JCOPTiX, TWP20H-532P) and a polarizing beam splitter (PBS, JCOPTiX, PBS25-532M) employed for continuous power adjustment. The beam was then shaped by a spatial filtering system (two convex lenses and a pinhole), passed through a collimation and homogenization converter, and finally incident on the SLM (Holoeye GAEA-2). The pre-designed large-FOV OAM-multiplexing hologram array was loaded onto the SLM. After modulation, the beam was resized by a two-lens system to match the dimensions of the hologram and sample. Reconstructed OAM patterns were captured at the Fourier plane of lens L5 using a charge-coupled device (CCD, Baslar, acA3088-57uc).

Since the OAM-multiplexing hologram design incorporates OAM-preserved holograms, as illustrated in Figure 4b, the OAM characteristics and mode orthogonality are well maintained at every pixel during reconstruction. Consequently, from a single OAM-multiplexing hologram array, different holographic images can be selectively decoded by switching forked nanograting array samples with opposite topological charges. Each view is uniquely linked to one diffraction order, thus enabling simultaneous observation of multiple perspectives. Specifically, loading the first hologram onto the SLM and switching the corresponding forked nanograting array enables reconstruction of four distinct large-FOV multiview3D scenes (Figure 4c). For example, sample 1 (*l*_*re*_ = −1) reconstructs digits (1–9), sample 2 (*l*_*re*_ = −5) reconstructs uppercase letters (A–I), while samples 3 and 4 reconstruct lowercase letters and Greek letters, respectively, with an angular FOV ranging from −14.66° to 14.66°. For the four reconstructed large field of view 3D images, we calculate its absolute efficiency (defined as the ratio of the energy of the reconstructed image at the OAM channel to the energy of input vortex beam), the absolute efficiency is 0.9 % at 532 nm. Notably, switching the hologram loaded onto the SLM while keeping the four forked nanograting array samples unchanged yields another set of four large-FOV multiview scenes. To demonstrate the performance of our work, Figure 4d presents image quality analysis of reconstructions from the first hologram using forked nanograting array with two different pixel sizes. Furthermore, to enhance the signal-to-noise ratio (SNR) of the constructed multiplexed holographic images, we applied a mode selective aperture array with periodicity derived from the sampling array in the hologram design. Compared with the target images, the reconstructed images achieved average SSIM values of 0.87 and 0.81, and average PSNR values of 14.54 dB and 12.88 dB, respectively. These results demonstrate that our method not only retains the dynamic modulation capability of SLM but also significantly extends the FOV of high-capacity multiview 3D display.

## 4 Conclusions

In summary, we propose a multiview 3D holographic display system based on OAM multiplexing and TPL-fabricated forked nanograting arrays, achieving simultaneous high-capacity information encoding and large-FOV display. The system integrates a 3 × 3 tiled OAM-multiplexed hologram array loaded on an SLM with a corresponding 3 × 3 forked nanograting array, enabling dynamic content switching and parallel multi-channel reconstruction. Each sub-hologram independently reconstructs four orthogonal OAM channels (*l*_en_ = 1, 5, −2, −5), and the grating’s multi-directional diffraction directs each channel to nine viewpoints, effectively expanding the FOV. The reconstructed images exhibit an average SSIM of ∼0.81 with negligible crosstalk, demonstrating high fidelity and reliability. The dual dynamic control mechanism provides flexible content refresh and OAM mode switching, further enhancing system versatility. A detailed comparison of the two dynamic content-switching schemes is provided in Supplementary Note 3 (Supplementary Information). This approach provides a new technical pathway for portable AR/VR devices, glasses-free multiview displays and secure optical communication. By integrating these two technologies, our approach addresses key challenges in AR/VR display development, offering a practical pathway toward next-generation devices that combine dynamic 3D visualization, large viewing angles, and compact design. Looking ahead, this technology could be combined with adaptive optics, super-resolution imaging and multi-mode light-field manipulation to achieve higher channel counts, wider FOVs and improved resolution for 3D displays, laying a solid foundation for next-generation high-performance multi-functional 3D display systems [2], [44], [45] and broadening applications in medical imaging, AR/VR and industrial inspection.

## 5 Methods

*Computational Platform*: All the calculations were performed on a personal desktop with an Intel(R) Core(TM) i5-10500 CPU @ 3.10 GHz, and 12 GB of RAM, running the Windows 10 for Workstations. The code was written, compiled, and run in the MATLAB R2022a software.

*Fabrication of the sample*: Both forked grating decoders in this paper were fabricated for experimentation using the nanofabrication process. By using the relation Δ*z* = *λ*Ф/2*π*Δ*n*, the phase value Φ of each pixel is accurately encoded into the relative height of the columnar structure, where *λ* denotes the wavelength of the target modulation beam and Δ*n* represents the refractive index difference between the photoresist and air. This ensures precise control over each diffraction order of the grating decoder. The details of the processing are available in Supplementary Note 4 (Supplementary Information).

*Statistical Analysis*: To quantitatively evaluate the similarity between the reconstructed holographic images and the target images, we employed two widely used image quality assessment metrics: the SSIM and the PSNR. SSIM measures perceptual similarity by jointly considering luminance, contrast, and structural information between two images. Its value ranges from 0 to 1, with values closer to 1 indicating higher similarity. The SSIM can be expressed as:

(6) $$SSIM\left(x,y\right)=\frac{\left(2\mu_{x}\mu_{y}+C_{1}\right)\left(2\sigma_{xy}+C_{2}\right)}{\left(\mu_{x}^{2}+\mu_{y}^{2}+C_{1}\right)\left(\sigma_{x}^{2}+\sigma_{y}^{2}+C_{2}\right)}$$

where *μ*_*x*_ and *μ*_*y*_ denote the mean intensity of images *x* and *y*, *σ*_*x*_^2^ and *σ*_*x*_^2^ represent their variances, *σ*_*xy*_ is the covariance, and *C*_1_ and *C*_2_ are small constants introduced to avoid instability. PSNR is derived from the mean squared error (MSE) and measures the pixel-wise fidelity between two images. It is defined as:

(7) $$PSNR=10\cdot \log_{10}\left(\frac{{MAX}_{I}^{2}}{MSE}\right)$$

where MAX_I_ is the maximum possible pixel value of the image. The MSE is given by:

(8) $$MSE=\frac{1}{MN}\overset{M}{\underset{i=1}{\sum }}{\overset{N}{\underset{j=1}{\sum }}\left[x\left(i,j\right)-y\left(i,j\right)\right]}^{2}$$

with *M* × *N* denoting the image size. A higher PSNR indicates better reconstruction fidelity. In this work, both SSIM and PSNR were employed to comprehensively assess the reconstruction quality under different experimental conditions, thereby providing an objective comparison of the imaging performance in large-FOV OAM-multiplexing holography.

## Supplementary Material

Supplementary Material Details
