# Supplementary material for: High-capacity multiview display with large viewing angle via orbital angular momentum-encoded nanograting arrays
Source: Nanophotonics. 2025 Nov 3;14(24):4341–50. doi: 10.1515/nanoph-2025-0433 (PMC12704489; doi:10.1515/nanoph-2025-0433)
Supplement: Supplementary file 1 — Supplementary Material Details [file j_nanoph-2025-0433_suppl_001.docx]

Supplementary Materials for

High-capacity multiview display with large viewing angle via orbital angular momentum-encoded nanograting arrays

*Yiqi Ye ^1,2^, Hang Su ^1,2^, Yuetian Jia ^1,2*^, Baoli Li ^1,2^, Min Gu^1,2^ and Xinyuan Fang ^1,2*^*

^1^School of Artificial Intelligence Science and Technology, University of Shanghai for Science and Technology, Shanghai, 200093, China.

^2^Institute of Photonic Chips, University of Shanghai for Science and Technology, Shanghai, 200093, China.

*Corresponding author. Email: yuetianjia@usst.edu.cn; [xinyuan.fang@usst.edu.cn](mailto:xinyuan.fang@usst.edu.cn)

**This PDF file includes:**

Supplementary Note 1 to 4

Supplementary Table S1

Supplementary Figures S1 to S3

**Supplementary Note 1: Validation of forked-nanograting array functionality via numerical simulations.**

A suite of numerical simulations was conducted to systematically validate the distinct roles of the forked-nanograting array in our system. The numerical analysis rests on three controlled comparisons. First, the transition from a single optical element (Supplementary Figure S1 (a)) to a full array architecture (Supplementary Figure S1 (d)) was shown to be fundamental for scaling the channel capacity, enabling the projection of nine independent data channels rather than a single channel. Second, by comparing an array of vortex phases with a diffractive nanograting array at an identical pixel size of 1 *µm* (Supplementary Figure S1 (b)), we confirmed that the diffractive structure is indispensable for effecting angular multiplexing, which spatially separates the channels and eliminates crosstalk. Third, analysis of two diffractive arrays with different pixel sizes (3.74 *µm* and 1 *µm,* Supplementary Figure S1 (c) and (d)) demonstrated that a reduction in pixel size directly enables a wider field of view, achieving a pronounced expansion from ±3.41° to ±15.1°. Together, these results provide multi-faceted evidence that our design successfully reconciles the core challenges of channel count, spatial isolation, and viewing angle.

**Supplementary Note 2: Method for calculating the field of view（FOV）of the holographic images.**

The FOV of the reconstructed image at a specific plane, which describes the angular extent of the image distribution on this plane, is given by the expression $\theta=2\tan^{-1}\left(D_{I}/2f\right)$^[1]^, where *D_I_* represents the size of the reconstructed image and *f* denotes the reconstruction distance from the hologram.

For the experimental results, we measured the total size of the reconstructed image recorded by the CCD as *D_I_* = 0.0268 *m* and the reconstruction distance of the holographic image as *f* = 0.05 *m*. The field of view was then calculated as:

$$\theta=2\tan^{-1}\left(D_{I}/2f\right)=2\tan^{-1}\left(0.0268/2\times0.05\right)=30^{\circ}$$

For the simulation results, the reconstructed image size and reconstruction distance were *D_I_* = 0.0119 *m* and *f* = 0.1 *m*, respectively, yielding a field of view of:

$\theta=2\tan^{-1}\left(D_{I}/2f\right)=2\tan^{-1}\left(0.0119/2\times0.1\right)=6.81^{\circ}$

**Supplementary Note 3: The key comparisons between two dynamic content switching schemes.**

1. SLM-based Switching: This approach enables dynamic image reconstruction while maintaining the encoded orbital angular momentum (OAM) sequence. The switching speed is primarily determined by the SLM's refresh rate (60 *Hz* for the HOLOEYE GAEA-2 device utilized in our experiments). The principal advantage of this method lies in its exceptional flexibility, permitting real-time reprogramming of arbitrary wavefronts without requiring mechanical adjustments or optical realignment. This characteristic makes it particularly suitable for applications demanding rapid content changes or interactive capabilities.

2. Nanograting array replacement: This method facilitates the reconstruction of information from different OAM channels within a single hologram through physical replacement of nanograting arrays. While the current manual replacement process results in slower switching speeds and necessitates optical realignment, each nanograting array functions as an independent optical decoder. This unique feature enables simultaneous reconstruction of completely different large-field scenes, representing a hardware-level expansion of system functionality that cannot be achieved through SLM updates alone.

In summary, these two switching modalities operate at different implementation levels: SLM updates provide software-level dynamic content switching, while nanograting replacement enables hardware-level system expansion and parallel output capability. The complementary nature of these approaches allows the system to achieve both rapid content refreshing within individual functional channels and robust multi-task processing capabilities through grating array replacement.

**Supplementary Note 4: The fabrication of micro-nano grating decoders using two-photon polymerization lithography(TPL).**

The fabrication of forked grating array was performed using a commercial lithography system (Photonic Professional GT, Nanoscribe GmbH) equipped with a 780 *nm*, 80 *MHz* femtosecond laser. The system employed an immersion galvanometer scanning mode configuration for printing, utilizing a high numerical aperture objective (Plan-Apochromat 63x/1.40 Oil DIC, Zeiss) and IP-Dip photoresist. The maximum heights corresponding to 2π phase modulation for the 532 *nm* channels were 1.064 *μm*. During processing, the slice distances (longitudinal laser movement steps) were set to 0.133 *μm*, corresponding to phase modulations of 1/4π for the 532 *nm* lasers. The lateral laser movement step was uniformly set to 0.1 *μm*. To achieve optimal results, the laser power and scanning speed were optimized to 50 *mW* and 10,000 *μm/s*. Post-printing, to ensure that the residual photoresist was removed while preserving the processed structure, the sample was immersed in propylene glycol 1-monomethyl ether 2-acetate for 30 minutes for development, followed by a 5-minute wash in Isopropanol. Finally, the sample was evaporated and dried in air.

**Supplementary Table S1:** Comparison of performance indexes of large field of view display.

|  | TPL (This work) | CMOS-based nanograting fabrication^[2]^ | Laser poling^[3]^ |
| --- | --- | --- | --- |
| Fabrication efficiency | Medium speed and low cost | Fast but costly | Slow speed and low cost |
| Scalability | Compatible with a variety of materials | High refractive index material | Ferroelectric crystal |
| Potential for practical device integration | Compact size and high integrability | Compact size and high integrability | High integration complexity |


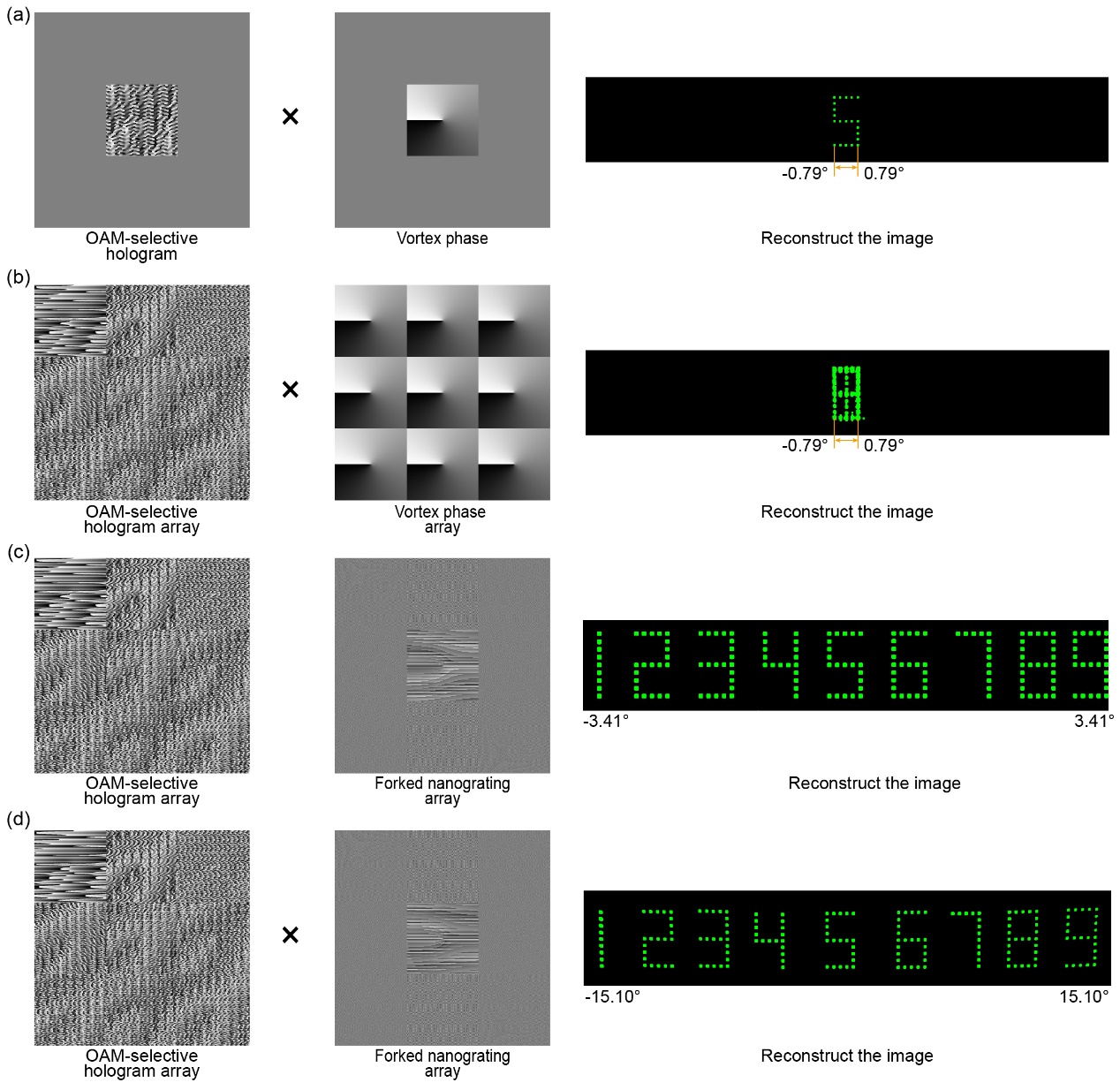


**Supplementary Figure S1.** Reconstruction results of OAM-selected hologram using different decoders. (a) Single vortex phase. (b) Vortex phase array. (c) Forked nanograting array with pixel size of 3.74 *μm*. (d) Forked nanograting array with pixel size of 1 *μm*.


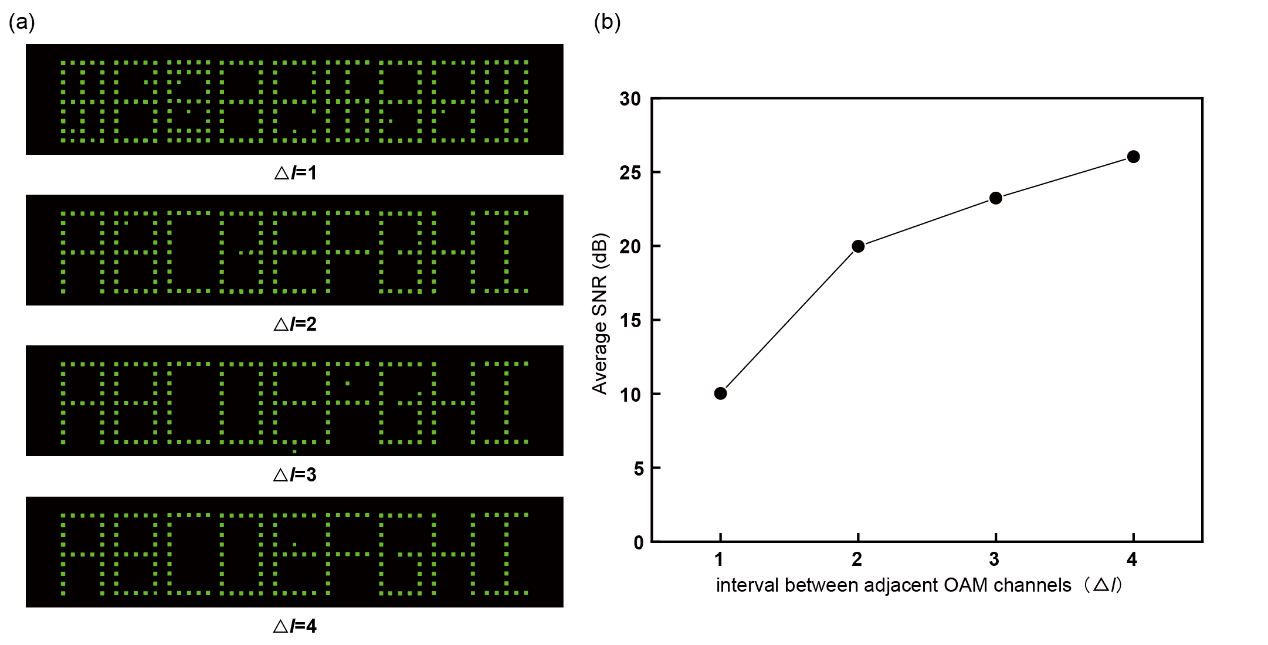


**Supplementary Figure S2.** The reconstruction holographic images encoded with different OAM channel intervals. (a) The holographic images reconstructed with different OAM intervals. (b) The relationship of the Average SNR with the adjacent OAM channel interval Δ*l*.


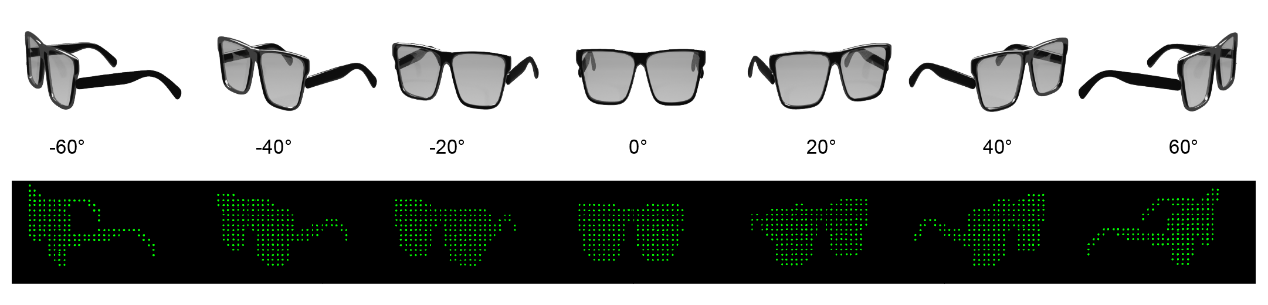


**Supplementary Figure S3.** A 3D “glasses” object reconstructed from seven viewing angles (-60°, -40°, -20°, 0°, 20°, 40°, 60°)

Reference:

[1] Z.-B. Fan et al., "Integral imaging near-eye 3D display using a nanoimprint metalens array," *eLight*, vol. 4, no. 1, p. 3, 2024.

[2] Z. Zhang et al., "Super‐Large Field‐of‐View, High‐Accurate and Real‐Time 3D Scene Reconstruction Based on Metasurface‐Enabled Structured Light," *Laser & Photonics Rev.*, vol. 19, no. 5, p. 2401120, 2025.

[3] X. Xu et al., "Large field-of-view nonlinear holography in lithium niobate," *Nano Lett.*, vol. 24, no. 4, pp. 1303-1308, 2024.
